# Plasmid transferability of KPC into a virulent K2 serotype *Klebsiella pneumoniae*

**DOI:** 10.1186/1471-2334-14-176

**Published:** 2014-03-31

**Authors:** Leung-Kei Kristopher Siu, David B Huang, Tom Chiang

**Affiliations:** 1 National Institute of Infectious Diseases and Vaccinology, National Health Research Institutes, Miaoli, Taiwan; 2 Graduate Institute of Basic Medical Science, China Medical University, Taichung, Taiwan; 3 Division of Infectious Diseases, Department of Medicine, Rutgers New Jersey Medical School, Newark, NJ, USA; 4 Department of Infectious Diseases, Veterans Affairs New Jersey Healthcare System, East Orange, NJ, USA; 5 Rutgers New Jersey Medical School, 185 South Orange Avenue, Newark, New Jersey 07101, USA

**Keywords:** *Klebsiella pneumoniae*, Virulence, Plasmid mediated resistance

## Abstract

**Background:**

KPC-producing carbapenem-resistant *Klebsiella pneumoniae* (CRKP) infections are associated with high mortality; however, their virulence determinants are not well defined.

**Methods:**

We investigated the virulence and plasmid transferability among KPC-containing *K. pneumoniae* isolates.

**Results:**

KPC-2 and -3 were successfully conjugated and retained by a virulent K2 *K. pneumoniae* recipient isolate. Antimicrobial susceptibility testing showed KPC-2 and -3 donor strains were resistant to more than four classes of antibiotics while the K2 isolate was only initially resistant to ampicillin. After conjugation of KPC-2 and -3, the K2 *K. pneumoniae* transconjugants became resistant to all beta-lactams. Additionally, the KPC K2 *K. pneumoniae* transconjugants continued to retain its high serum resistance and murine lethality.

**Conclusions:**

Conjugation and retainment of KPC by virulent K2 *K. pneumoniae* and the ability of the tranconjugants to maintain its high serum resistance and murine lethality after conjugation was demonstrated in this study. These findings are concerning for the potential of KPC-like genes to disseminate among virulent *K. pneumoniae* isolates.

## Background

In the Asia Pacific Rim, a pathogen that is well documented is the hypervirulent (hypermucoviscous) variant of *Klebsiella pneumoniae.* This pathogen causes unique clinical invasive syndromes of community-acquired *K. pneumoniae* infections including pyogenic liver abscess and a propensity for metastatic spread to distant sites in the absence of a history of hepatobiliary disease [1-3]. In Western countries, the predominant infections due to *K. pneumoniae* are still the “classic” strains. Infections caused by *K. pneumoniae* can result in serious and life threatening infections including pneumonia, urinary tract infections, intravascular line infections, soft tissue infections, intraabdominal infections and bacteremia. Because carbapenems are usually the last line of defense for the treatment of resistant Gram-negative pathogens, the development of carbapenemase-producing *K. pneumoniae* is of great concern. Carbapenem-resistant *K. pneumoniae* (CRKP) has become a major threat and burden to healthcare systems worldwide as most isolates are resistant to multiple classes and some to all classes of antibiotics making treatment difficult if not near impossible. However, most of these KPC-containing strains especially in the United States do not contain the virulence factors of the strains found in Asia resulting in a less invasive clinical presentation. Likewise, virulent strains of *K. pneumoniae* causing invasive disease that are usually found in Asia are not known to be multi-drug resistant let alone carbapenem-resistant. However, there have been recent separate reports of endemic KPC *K. pneumoniae* strains and hypervirulent invasive *K. pneumoniae* strains found in New Jersey. This study assesses the possibility of an invasive virulent K2 *K. pneumoniae* strain and its capability of acquiring KPC.

## Methods

### Strains in this study

The two most commonly found KPC strains (KPC-2 and -3) at the Veterans Affairs New Jersey Healthcare System (VA NJHCS) were used as donors. A virulent K2 serotype KP isolate from a liver abscess patient in New Jersey (University Hospital- Rutgers New Jersey Medical School) was chosen as the recipient [4]. These KPC KP donor isolates were acquired from the clinical microbiology laboratory at the VA NJHCS for this Institutional Review Board (IRB) approved study (MIRB 00937).

### Susceptibility testing

Antimicrobial susceptibility was determined by the broth microdilution method [5], according to the Clinical and Laboratory Standards Institute’s (CLSI) guidelines published in 2011. The following antimicrobial agents were used: cefazolin, amoxicillin/clavulanic acid, cefoxitin, cefotaxime, ceftazidime, imipenem, amikacin, gentamicin, ciprofloxacin, and trimethoprim-sulfamethoxazole.

### Pulsed-field gel electrophoresis

Pulsed-field gel electrophoresis (PFGE) was performed as previously described [6]. The resulting genomic DNA profiles, or fingerprints, were interpreted according to established guidelines [7,8].

### Confirmation of KPC production and KPC types by sequencing

All isolates selected were confirmed for KPC production by polymerase chain reaction (PCR). A gene-specific primer set, KPC-F (5′-ATG TCA CTG TAT CGC CGT CT-3′) and KPC-R (5′-TTT TCA GAG CCT TAC TGC CC-3′), was used to determine the presence of the KPC β-lactamase sequence [9]. The amplicons (893 bp) were sequenced using an automated sequencer by the Sanger method (ABI Prism 377 sequencer; Perkin-Elmer) [10]. The generated sequences were compared to the National Center for Biotechnology Information (NCBI) database at http://www.ncbi.nlm.nih.gov/blast/.

### Conjugation experiments

**Figure 1:**
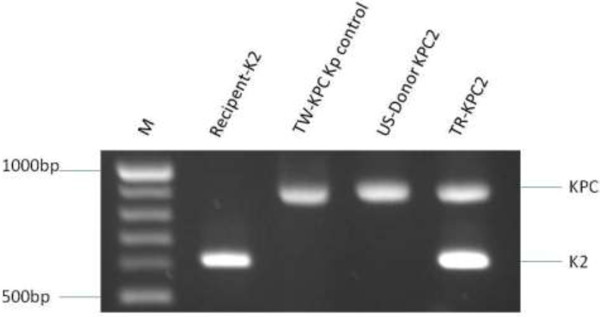
**Conjugation experiments of plasmids carrying *β*la**_**KPC**_**-2 and -3 from parental non-serotype K2 *K. pneumoniae *to recipient serotype K2 *K. pneumoniae*.** In this conjugation experiment, plasmids carrying *bla*KPC from KPC-positive *K. pneumoniae* (KPC-2 and -3) were successfully transferred into the invasive K2 *K. pneumoniae* recipient isolate (NJ-K2). Specifically, DNA extracted from the invasive K2 *K. pneumoniae* recipient isolate (NJ-K2) were positive for the *bla*KPC gene by PCR. Transconjugants, K2 *K. pneumoniae*::*β*la_KPC_, were obtained by *Klebsiella*-selective medium BIND supplemented with 2 µg/ml of imipenem. Colonies were then randomly selected from the plate and subjected for serotype K2 PCR. Colonies with PCR positive results on serotype determination were sub-cultured onto the same selective agar plate. Sub-culture of K2 *K. pneumoniae*:: *β*la_KPC_ occurred by inoculating onto MacConkey agar plates for 14 consecutive days. Lane 1, PCR verification of recipient serotype K2 isolate from New Jersey; Lane 2, Control KPC-2 from Taiwan; Lane 3, donor *K. pneumoniae* with *β*la_KPC-2_, Lane 4, transconjugants *K. pneumoniae*::*β*la_KPC-2_.

The transconjugant of the two most commonly found KPC strains (KPC-2 and -3) at the VA NJHCS, containing the KPC plasmid, were used as donors. The virulent K2 serotype *Klebsiella pneumoniae* (NJ-K2) isolate from a liver abscess patient in New Jersey was chosen as the recipient [4]. Inducing streptomycin resistance as a selection maker in the recipient for conjugation was performed according to previously described methods by Tasi *et al.*[11]. Briefly, the K2 serotype *K. pneumoniae* strain was grown in LB broth at 37°C to late-exponential growth phase and then spread on LB agar plates supplemented with 500 μg/ml streptomycin. The cells were incubated at 37°C and spontaneous mutants were obtained from colonies that arose within 3 days. This isolate possesses intrinsic resistance only to ampicillin and streptomycin. Mating procedure with KPC-2 and -3 containing KPC was then performed as previously described [12,13] except by using specific selection brilliant green agar-containing inositol-nitrate-deoxycholate (BIND) for *K. pneumoniae*[14]. Serotype K2/KPC transconjugants were selected using the *Klebsiella-*selective medium BIND supplemented with 2 μg/ml of imipenem and 50 μg/ml of streptomycin. The virulent K2 serotype KPC *K. pneumoniae* transconjugant was able to grow on the selective BIND medium with imipenem. Positive transconjugants were confirmed by positive PCR with specific primer sequences to serotype K2 [15] and KPC type β-lactamases genes [16]. Transconjugants from the conjugation experiments were subcultured onto MacConkey or Mueller Hinton agar (BBL) for 14 days to check for KPC plasmid stability in KPC strains (KPC-2 and -3) and *K. pneumoniae* after conjugation (Figure 1).

### PCR detection of virulence-associated genes rmpA and aerobactin, and serotypes K1, K2, and K5

PCR experiments to determine the presence of the specific genes for serotype K1, K2, and K5; *rmpA;* and the aerobactin gene were performed as previously described [15,17].

### Multilocus sequence typing (MLST)

MLST were performed according to Turton *et al*. [18]. Sequences of seven housekeeping genes were obtained for isolates from liver abscess patients and carriers. Sequence information was compared with that available from the MLST website (http://pubmlst.org/kpneumoniae/) developed by Keith Jolley. Alleles and sequence types (STs) were assigned accordingly. Sequences of any alleles that were not in the database were submitted to the curator and a new allele number obtained. A difference in two or more alleles was considered to indicate that the sequence types being compared were unrelated.

### Virulence studies

*In vitro* and *in vivo* assays to assess virulence were performed by serum resistance, neutrophil phagocytosis and mice lethality experiments. Neutrophil phagocytosis was performed as previously described [19]. The recipient serotype K2 *K. pneumoniae* (NJ-K2) isolated from a liver abscess was used as a control strain [4]. This strain is hypervirulent with a median lethal dose (LD50) < 10^2^ colony forming unit (CFU) and is highly phagocytic resistance (< 10% of phagocytic rate 15 minutes).

For the determination of LD50 in mice, pathogen-free, adult, 6-8-week-old, male BALB/c mice weighing 20–25 g were obtained from the National Laboratory Animal Center. This study was carried out in strict accordance with the recommendations in the Guide for the Care and Use of Laboratory Animals of the National Institutes of Health. All animal care procedures and protocols were approved by the Institutional Animal Care and Use Committee of the National Health Research Institute (NHRI-IACUC-096004-A). Tenfold serial dilutions of KP were made from a starting concentration of 10^6^ CFU, and the adult BALB/c mice had intraperitoneal injections with 0.1 ml of each concentration. Six mice were used as a sample population for each bacterial concentration [20]. Signs and symptoms of infection were observed for 14 days.

Survival of the inoculated mice was recorded, and the LD50 was calculated using SigmaPlot version 7.0 from SPSS Inc.

## Results

### Conjugation of plasmids of KPC-2 and -3 into serotype recipient K2 and antimicrobial susceptibility test of their transconjugants

In the conjugation experiment, plasmids carrying *bla*KPC from KPC-positive *K. pneumoniae* (KPC-2 and -3) were successfully transferred into the invasive K2 *K. pneumoniae* recipient isolate (NJ-K2) (Figure 1). Subculture of serotype K2 *K. pneumoniae::bla*KPC onto selective agar plate showed good growth in the selective BIND agar plate. No plasmid loss after 14 days of subculture onto the MacConkey or Mueller Hinton agar plate was observed for K2 *K. pneumoniae::bla*KPC indicating the stability of the *bla*KPC plasmid in *K. pneumoniae*. All strains were verified by PCR to ensure that no contamination occurred during each experiment for donor, recipient of serotype K2 *K. pneumoniae*, or transconjugants K2 *K. pneumoniae::bla*KPC.

The KPC-positive *K. pneumoniae* (KPC-2 and -3) isolates showed 100% resistance to piperacillin-tazobactam, cefazolin, cefotaxime, ceftazidime, ceftriaxone, ciprofloxacin, cefoxitin, gentamicin, imipenem and/or meropenem. All β-lactam resistance were maintained in transconjugants while change of susceptibilities from resistance to susceptible were observed in aminoglycosides and quinolones indicating non-plasmid encoding resistance in these classes of antibiotics (Table 1).

**Table 1:** Antimicrobial susceptibility test among donors of KPC- 2 or -3, recipient and transconjugant

**Antibiotics**	**Recipient K2**	**Donor**		**Transconjugant**	
		**KPC-2**	**KPC-3**	**KPC-2**	**KPC-3**
Ampicillin	≥32	≥32	≥32	≥32	≥32
Cefazolin	≤2	≥32	≥32	≥32	≥32
Cefoxitin	≤4	≥128	≥128	≥128	≥128
Aztreonam	≤1	≥32	≥32	≥32	≥32
Cefotaxime	≤1	32	32	8	8
Ceftazidime	≤1	≥32	≥32	≥32	≥32
Cefepime	≤1	≥32	16	16	8
Ertapenem	≤0.25	≥8	≥8	≥8	≥8
Imipenem	0.5	≥8	≥8	≥8	≥8
Meropenem	≤0.25	≥8	≥8	≥8	≥8
Doripenem	≤0.12	≥4	≥4	≥4	≥4
Colistin	1	≤0.5	≥4	≤1	1
Tigecycline	≤0.25	1	≤0.25	≤0.25	≤0.25
Gentamicin	≤1	≤1	≥16	≤1	≤1
Amikacin	≤4	16	32	≤4	≤4
Ciprofloxacin	0.12	≥4	≥4	≤0.06	0.12
Levofloxacin	≤0.5	≥8	≥8	≤0.5	≤0.5
SXT*	≤0.5	≥4	≥4	≥4	≤0.5

*Cotrimoxazole.

### Detection of virulence-associated determinants and mice lethality experiments

**Figure 2:**
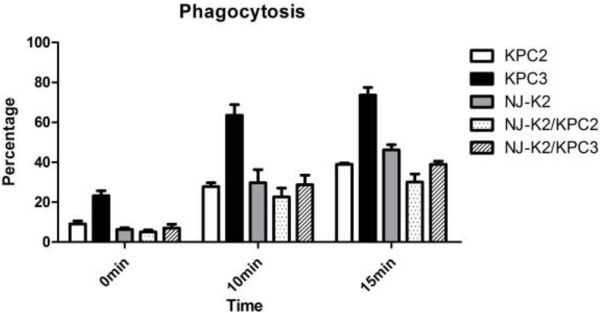
Neutrophils phagocytosis assays among Isolates of KPC-2 and -3, serotype K2 recipient and their transconjugants.

**Figure 3:**
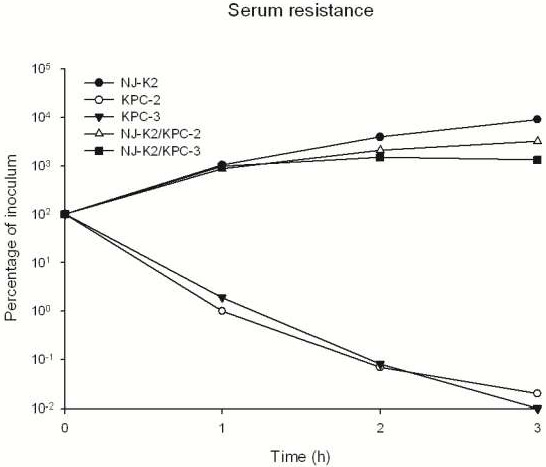
Serum resistance assays among Isolates of KPC-2 and -3, serotype K2 recipient and their transconjugants.

Detection of K1, K2, and K5 virulence-associated serotypes, and *rmpA* and aerobactin genes, revealed that KPC donor isolates were non-Kl, -K2, or -K5 serotypes and did not carry *rmpA* and aerobactin virulence genes. MLST showed that both donor isolates were ST-258 and recipient serotype K2 was ST-65. Transconjugants carrying plasmid encoding KPC-2 or 3 showed an increasing resistance to neutrophil phagocytosis in comparing to donor strains which carry KPC-2 or -3 carbapenemase (Figure 2). In assessing compliment killing by serum resistance assay, all the transconjugants were resistant to serum killing as recipient serotype K2 *K. pneumoniae* (Figure 3).

**Figure 4:**
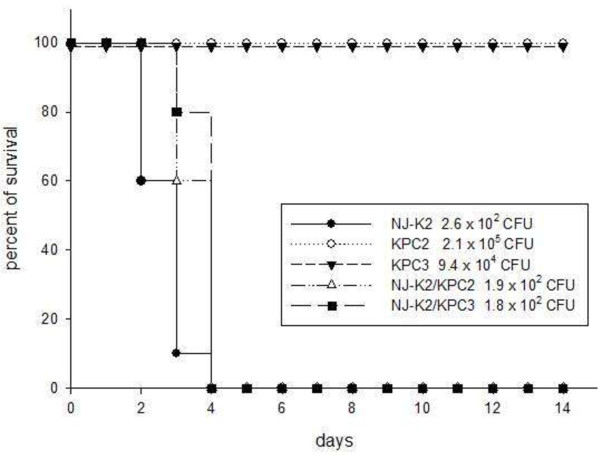
**Mice lethality test among Isolates of KPC-2 and -3, serotype K2 recipient and their transconjugants.** The survivals after inoculation of *K. pneumonia*e were counted over 14 days. Data points represent the percentage of mice surviving in each experimental group over time. (n = 6 mice per strain, pooled from two separate experiments).

Murine lethality studies by IP injection showed that LD50 were > 10^6^ CFU for the KPC *K. pneumoniae* isolates prior to conjugation, indicating low virulence. In contrast, LD50 of the transconjugant strain was < 10^2^ CFU after conjugation while retaining KPC and K2 genes [confirmed by PCR (Figure 4)]. In order to confirm the stability of KPC-2 or 3 in transconjugants, PCR detection for serotype K2 and KPC-2 or 3 were used during the infection period. PCR of serotype K2 and KPC-2 or 3 were detected on day 2 and day3 of isolation indicating the KPC-2 or -3 plasmids were stable in the transconjugants during infection.

## Discussion

KPC *K. pneumoniae* was first reported in the United States in 2001. Since its first report, KPC *K. pneumoniae* has become an increasingly prevalent pathogen and is now endemic in the Northeastern United States. Its spread has been well documented worldwide. According to data from the National Nosocomial Infections Surveillance System Report and the National Healthcare Safety Network, the proportion of Enterobacteriaceae that are carbapenem-resistant has increased from 1.2% in 2001 to 4.2% in 2011. At the VA NJHCS and its affiliated University Hospital (Rutgers New Jersey Medical School), *K. pneumoniae* is now the second most prevalent Gram-negative pathogen with carbapenem resistance rates of 18% and 12% respectively (2012 VA NJHCS and University Hospital antibiograms). Most of the CRKP isolates found at these two hospitals are multi-drug resistant if not pan resistant to all available antibiotics. Not surprisingly, mortality rates of up to 50% have been reported in CRKP bacteremia cases due to the lack of effective antibiotics. Fortunately, CRKP has yet to be associated with community acquisition.

Invasive KP was initially thought of as a regionally distributed disease found in the Asia-Pacific Rim. However, it is now been reported worldwide as an emerging infectious disease [21]. Its virulence determinants and phagocytic inhibition has been well studied [22]. Unlike invasive *K. pneumoniae*, virulence and plasmid transmissibility of KPC *K. pneumoniae* have not been well investigated. Our previous study of virulence in KPC *K. pneumoniae* showed that in 60 unique CRKP, none had the virulent serotypes K1, K2, or K5 nor the virulence factors of rmpA or aerobactin lending to the assumption that high rates of morbidity and mortality due to KPC *K. pneumoniae* are most likely due to its antibiotic resistance properties and not its virulence.

This study showed that the combination of an increased virulent strain of *K. pneumoniae* acquiring KPC and its associated multidrug associated genes can be created. Conjugation and retainment of KPC by virulent K2 *K. pneumoniae* and the ability of the tranconjugants to maintain its high serum resistance and lethality after conjugation were possible. The ramifications of having a virulent, multi-drug resistant, KPC-containing *K. pneumoniae* would be disastrous on healthcare systems [23]. Not surprisingly, with continued indiscriminate use of antibiotics worldwide, KPC-producing *K. pneumoniae* has been detected in China with the predominate clone being ST11 which is unrelated to the current ST258 clone found commonly in KPC *K. pneumoniae* found in the United States. With the continued worldwide dissemination of KPC, it is likely a matter of time before the virulent Asian strains of KP will begin to express KPC carbapenem resistance.

## Conclusions

As demonstrated in our study, further dissemination of the KPC plasmid into virulent types of *K. pneumoniae* cannot be excluded in the future. Efforts to assess the factors that contribute to attributable mortality in KPC *K. pneumoniae* infections should be continued, including the study of other virulence determinants, antibiotic resistance, host responses, and their relationship with clinical characteristics and outcomes.

## Competing interests

The authors declare that they have no competing interest.

## Authors’ contributions

KS, DBH, and TC made substantial contributions to conception and design, acquisition of data, and analysis and interpretation of data. KS, DBH, and TC were involved in drafting the manuscript, revising it critically for important intellectual content. All authors read and approved the manuscript.

## Pre-publication history

The pre-publication history for this paper can be accessed here:

http://www.biomedcentral.com/1471-2334/14/176/prepub

## References

[B1] Cheng DL Liu YC Yen MY Liu CY Wang RS Septic metastatic lesions of pyogenic liver abscess. Their association with Klebsiella pneumoniae bacteremia in diabetic patients Arch Intern Med 1991 14 8 1557 1559 10.1001/archinte.1991.00400080059010 1872659

[B2] Liu YC Cheng DL Lin CL Klebsiella pneumoniae liver abscess associated with septic endophthalmitis Arch Intern Med 1986 14 10 1913 1916 10.1001/archinte.1986.00360220057011 3532983

[B3] Wang JH Liu YC Lee SS Yen MY Chen YS Wann SR Lin HH Primary liver abscess due to Klebsiella pneumoniae in Taiwan Clin Infect Dis 1998 14 6 1434 1438 10.1086/516369 9636876

[B4] Rivero A Gomez E Alland D Huang DB Chiang T K2 serotype Klebsiella pneumoniae causing a liver abscess associated with infective endocarditis J Clin Microbiol 2010 14 2 639 641 10.1128/JCM.01779-09 20007381 PMC2815605

[B5] Ambrozic J Ostroversnik A Starcic M Kuhar I Grabnar M Zgur-Bertok D Escherichia coli CoIV plasmid pRK100: genetic organization, stability and conjugal transfer Microbiology 1998 14 Pt 2 343 352 9493372 10.1099/00221287-144-2-343

[B6] D’Agata EM Gerrits MM Tang YW Samore M Kusters JG Comparison of pulsed-field gel electrophoresis and amplified fragment-length polymorphism for epidemiological investigations of common nosocomial pathogens Infect Control Hosp Epidemiol 2001 14 9 550 554 10.1086/501950 11732783

[B7] McDougal LK Steward CD Killgore GE Chaitram JM McAllister SK Tenover FC Pulsed-field gel electrophoresis typing of oxacillin-resistant Staphylococcus aureus isolates from the United States: establishing a national database J Clin Microbiol 2003 14 11 5113 5120 10.1128/JCM.41.11.5113-5120.2003 14605147 PMC262524

[B8] Tenover FC Arbeit RD Goering RV Mickelsen PA Murray BE Persing DH Swaminathan B Interpreting chromosomal DNA restriction patterns produced by pulsed-field gel electrophoresis: criteria for bacterial strain typing J Clin Microbiol 1995 14 9 2233 2239 7494007 10.1128/jcm.33.9.2233-2239.1995 PMC228385

[B9] Bradford PA Bratu S Urban C Visalli M Mariano N Landman D Rahal JJ Brooks S Cebular S Quale J Emergence of carbapenem-resistant Klebsiella species possessing the class A carbapenem-hydrolyzing KPC-2 and inhibitor-resistant TEM-30 beta-lactamases in New York City Clin Infect Dis 2004 14 1 55 60 10.1086/421495 15206053

[B10] Sanger F Air GM Barrell BG Brown NL Coulson AR Fiddes CA Hutchison CA Slocombe PM Smith M Nucleotide sequence of bacteriophage phi X174 DNA Nature 1977 14 5596 687 695 10.1038/265687a0 870828

[B11] Tsai YK Fung CP Lin JC Chen JH Chang FY Chen TL Siu LK Klebsiella pneumoniae outer membrane porins OmpK35 and OmpK36 play roles in both antimicrobial resistance and virulence Antimicrob Agents Chemother 2011 14 4 1485 1493 10.1128/AAC.01275-10 21282452 PMC3067157

[B12] Siu LK Ho PL Yuen KY Wong SS Chau PY Transferable hyperproduction of TEM-1 beta-lactamase in Shigella flexneri due to a point mutation in the pribnow box Antimicrob Agents Chemother 1997 14 2 468 470 9021210 10.1128/aac.41.2.468 PMC163732

[B13] Wang M Tran JH Jacoby GA Zhang Y Wang F Hooper DC Plasmid-mediated quinolone resistance in clinical isolates of Escherichia coli from Shanghai, China Antimicrob Agents Chemother 2003 14 7 2242 2248 10.1128/AAC.47.7.2242-2248.2003 12821475 PMC161834

[B14] Yeh KM Lin JC Yin FY Fung CP Hung HC Siu LK Chang FY Revisiting the importance of virulence determinant magA and its surrounding genes in Klebsiella pneumoniae causing pyogenic liver abscesses: exact role in serotype K1 capsule formation J Infect Dis 2010 14 8 1259 1267 10.1086/606010 19785524

[B15] Turton JF Baklan H Siu LK Kaufmann ME Pitt TL Evaluation of a multiplex PCR for detection of serotypes K1, K2 and K5 in Klebsiella sp. and comparison of isolates within these serotypes FEMS Microbiol Lett 2008 14 2 247 252 10.1111/j.1574-6968.2008.01208.x 18507682

[B16] Chiu SK Wu TL Chuang YC Lin JC Fung CP Lu PL Wang JT Wang LS Siu LK Yeh KM National surveillance study on carbapenem non-susceptible Klebsiella pneumoniae in Taiwan: the emergence and rapid dissemination of KPC-2 carbapenemase PLoS One 2013 14 7 e69428 10.1371/journal.pone.0069428 23894478 PMC3722148

[B17] Siu LK Fung CP Chang FY Lee N Yeh KM Koh TH Ip M Molecular typing and virulence analysis of serotype K1 Klebsiella pneumoniae strains isolated from liver abscess patients and stool samples from noninfectious subjects in Hong Kong, Singapore, and Taiwan J Clin Microbiol 2011 14 11 3761 3765 10.1128/JCM.00977-11 21900521 PMC3209116

[B18] Turton JF Englender H Gabriel SN Turton SE Kaufmann ME Pitt TL Genetically similar isolates of Klebsiella pneumoniae serotype K1 causing liver abscesses in three continents J Med Microbiol 2007 14 Pt 5 593 597 17446279 10.1099/jmm.0.46964-0

[B19] Lin JC Chang FY Fung CP Xu JZ Cheng HP Wang JJ Huang LY Siu LK High prevalence of phagocytic-resistant capsular serotypes of Klebsiella pneumoniae in liver abscess Microbes Infect 2004 14 13 1191 1198 10.1016/j.micinf.2004.06.003 15488738

[B20] Yigit H Queenan AM Anderson GJ Domenech-Sanchez A Biddle JW Steward CD Alberti S Bush K Tenover FC Novel carbapenem-hydrolyzing beta-lactamase, KPC-1, from a carbapenem-resistant strain of Klebsiella pneumoniae Antimicrob Agents Chemother 2001 14 4 1151 1161 10.1128/AAC.45.4.1151-1161.2001 11257029 PMC90438

[B21] Pournaras S Zarkotou O Poulou A Kristo I Vrioni G Themeli-Digalaki K Tsakris A A combined disk test for direct differentiation of carbapenemase-producing enterobacteriaceae in surveillance rectal swabs J Clin Microbiol 2013 14 9 2986 2990 10.1128/JCM.00901-13 23843486 PMC3754636

[B22] Siu LK Yeh KM Lin JC Fung CP Chang FY Klebsiella pneumoniae liver abscess: a new invasive syndrome Lancet Infect Dis 2012 14 11 881 887 10.1016/S1473-3099(12)70205-0 23099082

[B23] Li W Sun G Yu Y Li N Chen M Jin R Jiao Y Wu H Increasing occurrence of antimicrobial-resistant hypervirulent (hypermucoviscous) Klebsiella pneumoniae isolates in China Clin Infect Dis 2014 14 2 225 232 10.1093/cid/cit675 24099919

